# COPD Assessment Test Variability as a Predictor of Exacerbations in Patients with COPD: A Prospective Study Using Virtual Assistant Monitoring

**DOI:** 10.3390/arm94050064

**Published:** 2026-09-07

**Authors:** Cristina Aljama, Robson Aparecido Prudente, Ane Lopez-Gonzalez, María Sáez, Suzana E. Tanni, Júlia Sampol, Núria Rodés, Yesly Esthepannie Carlos, Mercedes Pallero, Sergi Martí, Gerard Orriols, Marian Ramon, Galo Granados, Cristina Esquinas, Marc Miravitlles, Miriam Barrecheguren

**Affiliations:** 1 Pneumology Department, Hospital Universitari Vall d’Hebron, Vall d’Hebron Institut de Recerca (VHIR), Vall d’Hebron Barcelona Hospital Campus, 08035 Barcelona, Spain; cris.aljama94@gmail.com (C.A.); anelopez95@gmail.com (A.L.-G.); maria.saez@vallhebron.cat (M.S.); julia.sampol@vallhebron.cat (J.S.); nuria.rodes@vallhebron.cat (N.R.); yeslyesthepannie.carlos@vallhebron.cat (Y.E.C.); mercedes.pallero@vallhebron.cat (M.P.); sergiomanuel.marti@vallhebron.cat (S.M.); g.orriolstorras@gmail.com (G.O.); ma.ramon@vhir.org (M.R.); galodavid.granados@vallhebron.cat (G.G.); marcm@separ.es (M.M.); 2 School of Medicine, São Paulo State University (UNESP), Botucatu 18618-687, Brazil; robsonapp@gmail.com (R.A.P.);; 3 Department of Public Health Nursing, Mental Health and Maternal and Child Health, Faculty of Nursing, University of Barcelona, 08036 Barcelona, Spain; crise4@hotmail.com

**Keywords:** chronic obstructive pulmonary disease, COPD assessment test, exacerbations, symptom variability, digital health monitoring

## Abstract

**Highlights:**

**What are the main findings?**

- Weekly increases in COPD Assessment Test (CAT) scores were independently associated with a higher risk of COPD exacerbation, with the risk increasing progressively as CAT scores worsened.
- A short-term increase of ≥3 CAT points provided the best discriminative threshold for exacerbation events.

**What are the implications of the main findings?**

- Short-term monitoring of CAT scores may help to detect COPD exacerbations before clinical deterioration.
- Incorporating serial CAT assessment into remote monitoring programs could facilitate earlier intervention and improve exacerbation management.

**Abstract:**

Although short-term CAT score variability has been associated with COPD exacerbations, evidence supporting its integration into virtual assistant-based monitoring remains limited. The aim of this study was to assess the short-term variability in CAT scores using a virtual management tool in patients with COPD and to examine its relationship with the prediction of exacerbations. A prospective observational single-center study was conducted in patients aged >35 with COPD and an exacerbator phenotype (≥2 outpatient exacerbations or ≥1 hospitalization in the previous year). Follow-up was performed weekly, including the CAT questionnaire, through calls were conducted by the virtual assistant Tucuvi Health Manager^®^ during a period of one year. A total of 106 patients were included; 64.2% were male, with a mean (SD) age of 66.8 (7.9) years and a median FEV_1_(%) of 38.8 (30.1–45.0). The mean follow-up was 25 weeks [IQR 20–35], during which 73 (69%) patients experienced ≥1 exacerbation, accounting for 192 episodes (26% of which required hospitalization). A total of 2550 CAT scores were collected. The mean baseline CAT score was 17.9 (7.6), and scores increased significantly during exacerbations compared with non-exacerbation periods and according to exacerbation severity. Each one-point increase in absolute CAT score was associated with an 11.1% increase in the odds of exacerbation (OR: 1.11; 95% CI: 1.08–1.13), while a weekly variation of ≥2 points (MCID) was associated with more than sixfold higher odds (OR: 6.30; 95% CI: 4.28–9.27). The optimal cut-off for CAT change in the prediction of exacerbation risk was 3 points with a ROC curve of 0.773. Weekly deterioration in CAT scores was associated with increased exacerbation risk in patients with COPD. Short-term increases in CAT may serve as clinically useful warning signals for exacerbation.

## 1. Introduction

Chronic Obstructive Pulmonary Disease (COPD) is characterized by progressive airway obstruction with frequent episodes of exacerbation that significantly worsen the quality of life of patients, increase the risk of serious complications and, in many cases, contribute to premature mortality [1,2,3,4]. The impact of exacerbations is not only clinical, but also economic. The management of acute episodes requires a high consumption of healthcare resources, including hospitalizations, intensive pharmacological treatment and intensive medical follow-up, which considerably raises the costs associated with the treatment of COPD [5]. Consequently, strategies to reduce readmission rates are a priority in mitigating the burden of COPD [6,7].

However, exacerbations are often underdiagnosed or recognized with great delay due to several factors, including patients’ inability to differentiate between acute episodes and symptom fluctuations, delays in seeking medical care and disparities in the perception of dyspnea [8]. This is concerning, as early treatment enhances recovery, reduces hospitalization risk, and improves health-related quality of life [9]. A key objective in COPD management is, therefore, the early identification and treatment of exacerbations to prevent subsequent episodes [10].

To assess symptom control and disease burden in patients with COPD, tools such as the COPD Assessment Test (CAT) questionnaire have been developed [11]. The CAT has demonstrated excellent correlation with other validated quality of life questionnaires [12,13] and the CAT score may be a reliable predictor of future exacerbations [14]. Recent studies showed that higher CAT scores were associated with an increased risk of exacerbations [15], and longitudinal changes in CAT scores can aid in early detection of exacerbations [16].

Close monitoring of patients with COPD, particularly those with recurrent exacerbations, remains a major challenge in disease management. Digital health technologies, including artificial intelligence-based systems and virtual case managers, may facilitate longitudinal symptom assessment, support timely clinical intervention and optimize the use of healthcare resources [17,18,19,20]. Voice-based virtual assistants, such as the Tucuvi Health Manager^®^, have previously demonstrated feasibility and acceptance for the remote monitoring of patients with other respiratory conditions, including patients with COVID-19, following hospital discharge [21]. However, evidence regarding the combined use of such technologies and serial CAT assessments to identify short-term changes associated with an increased risk of COPD exacerbation remains limited. Evaluating this approach may therefore help determine whether digitally administered CAT monitoring can provide clinically useful warning signals in patients at high risk of exacerbation.

The aim of this study was to evaluate weekly CAT score variability, collected through a voice-based virtual monitoring assistant, and its association with exacerbations in patients with COPD and an exacerbator phenotype.

## 2. Materials and Methods

### 2.1. Design of the Study

This was an observational and prospective study conducted at the Vall d’Hebron University Hospital (Barcelona, Spain) from March 2024 to March 2025 using the Tucuvi Health Manager (THM) digital tool (V2025). THM is a cloud-based Class I medical device developed by Tucuvi Care S.L. (Madrid, Sapin). It enables remote patient monitoring through a voice-based virtual assistant that interacts with patients via automated telephone calls, without requiring installation of any device or internet connection [21].

Patients received weekly calls from the virtual assistant and selected their preferred day and time slot for the call. Calls were made from Monday to Friday, between 9:00 AM and 8:00 PM. The assistant assessed general and COPD-related symptom questions and generated a data matrix within THM, a software-based medical device that collects patient-reported symptoms and signs. Based on the responses, alerts of varying priority levels were triggered, requiring review by the responsible healthcare team. This system allowed providers to focus on patients experiencing significant changes in their clinical status or biometric data. After reviewing the records, if deemed necessary, a medical team member contacted the patient and, if required, arranged for an intervention or additional visit.

The project was developed in accordance with the principles established in the Declaration of Helsinki (1964), the Council of Europe Convention on Human Rights and Biomedicine (1997), and the UNESCO Universal Declaration on the Human Genome and Human Rights, as well as the requirements established in Spanish legislation on biomedical research, bioethics, and personal data protection (Organic Law 3/2018 on Personal Data Protection and the General Data Protection Regulation (GDPR) 2016/679). The study was approved by the Ethics Committee and all patients provided written informed consent.

### 2.2. Participants

Patients diagnosed with COPD (over 35 years old, smokers or ex-smokers with a cumulative smoking history of at least 10 pack-years, with post-bronchodilator spirometry showing a forced expiratory volume in 1 s (FEV_1_)/forced vital capacity (FVC) < 0.7) and defined as exacerbators, with at least two or more outpatient exacerbations or one or more hospitalizations due to COPD exacerbation in the last 12 months, were included. Patients were recruited consecutively in the pneumology outpatient clinic or in the hospital ward.

Patients without the ability to answer phone calls, without a caregiver who could answer them, or suffering from a terminal illness other than COPD with a life expectancy of less than one year were excluded.

### 2.3. Measurements

At baseline, the following data were collected: anthropometric and sociodemographic data, the respiratory treatment used by the patient and the number of exacerbations in the previous year. The degree of dyspnea was evaluated with the modified Medical Research Council (mMRC) scale [22], and comorbidities were evaluated with the Charlson Index [23]. The values of forced spirometry, carbon monoxide diffusion capacity (DLCO) and blood test data were collected. Spirometry-predicted values were calculated using the 2012 Global Lung Function Initiative multi-ethnic reference equations [24]. The use of rescue medication, corticosteroids, and/or antibiotics, as well as emergency room visits, were also recorded. Emphysema was defined by radiological evidence on chest computed tomography of areas of abnormally low attenuation caused by pulmonary parenchymal destruction [25]. Chronic bronchitis was defined as cough and sputum production for at least three months per year during two consecutive years, after excluding other causes [26]. Obstructive sleep apnea was defined as a previously established diagnosis based on polysomnography or a technically adequate home sleep apnea test showing an apnea–hypopnea index or respiratory event index of ≥15 events/h, irrespective of symptoms, or ≥5 events/h in the presence of compatible symptoms, clinical features or associated comorbidities [27].

During follow-up, the variables collected by the virtual assistant included: patient identification, general well-being, increased sputum production or changes in sputum color, CAT score, daily physical activity, dyspnea corresponding to an mMRC grade ≥2, fever, self-reported oxygen saturation, use and daily duration of long-term home oxygen therapy, regular maintenance treatment, use of rescue medication, perceived need for emergency department assessment, and any additional observations reported by the participant. If the participant did not answer the initial call, up to two additional attempts were made the same day.

The CAT score collected weekly via the virtual assistant served as the primary exposure variable, enabling continuous monitoring of patient-reported symptoms and health status changes over time. CAT scores were assessed prospectively at weekly intervals. When an exacerbation was reported, its temporal relationship with the corresponding CAT assessment was determined according to the timing of the patient-reported event. Because exacerbation onset could occur between weekly assessments, CAT measurements could reflect symptom status before, during, or after the onset of an exacerbation. For CAT variability analyses, weekly CAT change was calculated within each patient as the difference between the current CAT score and the score obtained at the preceding weekly assessment. CAT assessments that were not completed were treated as missing and were not imputed, and weekly CAT changes were calculated only when CAT scores were available for two consecutive weekly assessments. Specifically, the CAT is a questionnaire composed of eight items addressing the presence of cough, sputum, chest tightness, breathlessness, limitations in home activities, loss of confidence at home, sleep quality, and energy levels. For each item, the patient selects one response option scored from 0 to 5. The individual item scores are summed to yield a total score reflecting the clinical impact of COPD. The results are classified according to their clinical significance as follows: low (0–10 points), medium (11–20 points), high (21–30 points), and very high (31–40 points). A difference of at least 2 points in the CAT score represents the minimum clinically important difference (MCID), reflecting the smallest change perceived as clinically meaningful by patients [11,28].

Additionally, exacerbations experienced by the participants during follow-up were recorded. Exacerbations were categorized into mutually exclusive groups: mild, when only treatment with short-acting beta2-agonist (SABA) and/or short-acting muscarinic antagonist (SAMA) was increased; outpatient-treated, when corticosteroids and/or antibiotics were prescribed in an outpatient setting; emergency department, when urgent care was required without hospitalization; and hospital admission, when hospitalization was required [26]. Data on exacerbations were collected through patient self-reporting during weekly calls with the virtual assistant and confirmed by review of electronic medical records. This approach enabled longitudinal monitoring and precise classification of exacerbation events, supporting the analysis of their association with CAT score trajectories.

### 2.4. Statistical Analysis

Descriptive statistics were used to summarize the characteristics of the study participants. Continuous variables were presented as mean (standard deviation, SD) or median (interquartile range, IQR), as appropriate.

To analyze longitudinal changes in CAT scores over time and their relationship with exacerbations, linear mixed-effects models were used. In these models, CAT score was treated as a continuous dependent variable, and time, exacerbation type, and the interaction between time and exacerbation type were included as fixed effects. A random intercept and slope for time were included for each participant (ID) to account for repeated measures and inter-individual variability in symptom trajectories.

To evaluate the association between CAT score variability and the risk of exacerbation, generalized estimating equations with a binomial distribution and logit link function were applied, accounting for repeated observations within individuals. Absolute CAT score was first analyzed as a continuous predictor. Subsequently, weekly CAT change, calculated as the difference between the current and preceding weekly CAT scores, was categorized into three groups: ≥2-point increase, no change (−1 to +1 points), and ≥2-point decrease, based on the established MCID to facilitate clinical interpretation. CAT assessments that were not completed were treated as missing and were not imputed. Weekly CAT change was calculated only when CAT scores were available for two consecutive weekly assessments; no change score was calculated when either assessment was missing. Analyses were therefore based on available complete observations. The analyses implicitly assumed that missing CAT assessments were unrelated to unobserved outcomes conditional on the observed data; however, the missing-data mechanism was not formally tested.

Receiver operating characteristic (ROC) curve analysis was performed to evaluate the discriminative ability of weekly CAT score variation in relation to exacerbation events. Because multiple CAT assessments were obtained from each participant, a patient-level cluster bootstrap sensitivity analysis with 5000 resamples was performed to assess the impact of within-patient clustering on the ROC estimate.

A significance level of 5% was applied for all tests. Statistical analyses were performed using Jamovi version 2.7.12.0 (2025) (The Jamovi Project, Sydney, Australia) and SPSS version 20.0 (2011) (IBM Corp., Armonk, NY, USA).

Because an a priori sample size calculation was not performed, a post hoc power analysis was conducted using G*Power version 3.1.9.7. Considering a total sample size of 106 participants, an alpha level of 0.05, and an observed variance ratio of 0.68, the achieved statistical power was 86.5%.

## 3. Results

### 3.1. Patients’ Characteristics and Exacerbations

**Figure 1:**
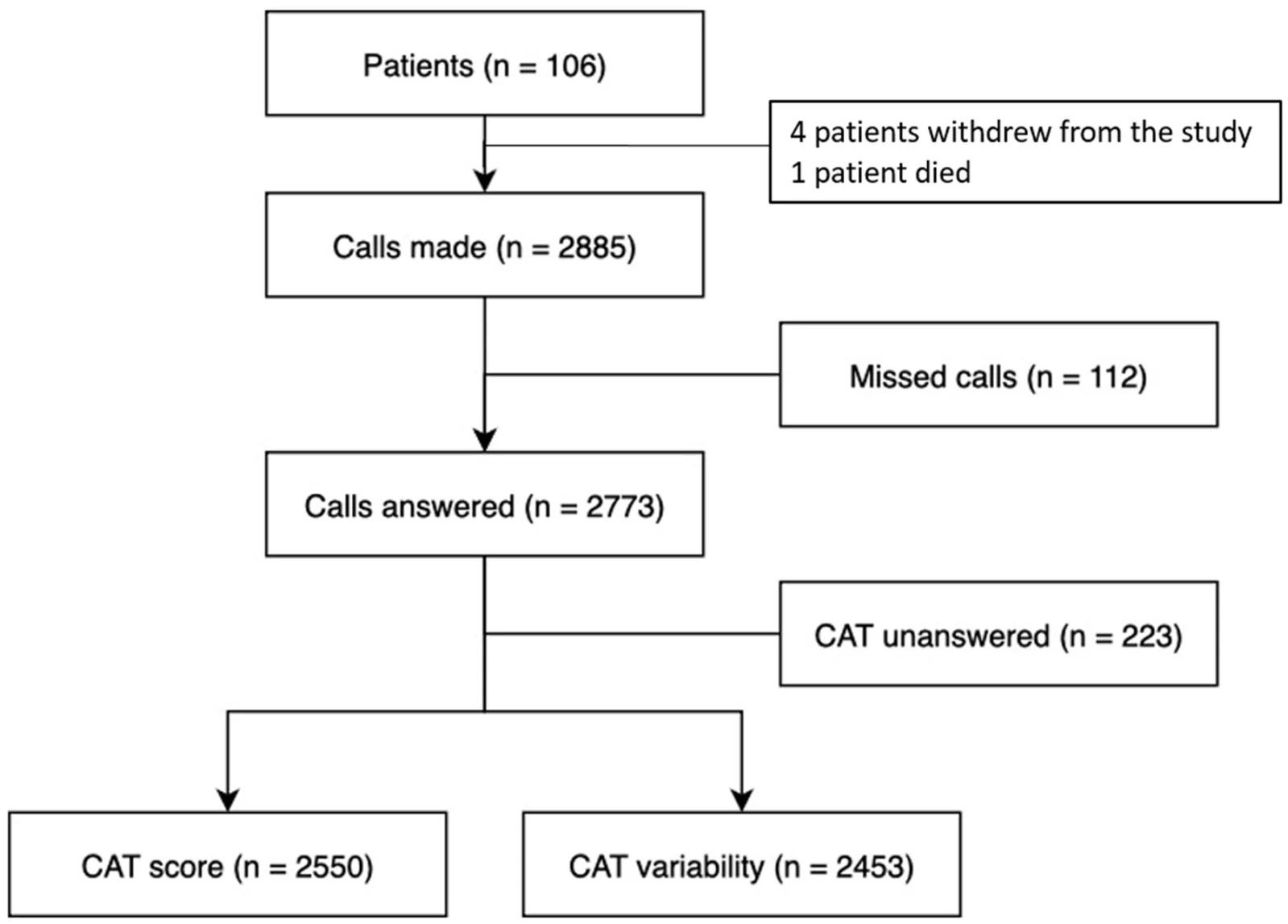
Study flow chart: patient calls and CAT completion.

A total of 106 individuals were included in the study. Of them, 68 (64.2%) were male, with a mean age of 66.8 (SD 7.9) years and a mean forced expiratory volume in one second (FEV_1_%) of 38.8% (IQR 30.1–45.0) predicted. At baseline, the average CAT score was 17.9 (SD 7.6). The remaining characteristics of the participants are presented in Table 1. During follow-up, 73 patients (69%) experienced one or more exacerbations, totaling 192 episodes. These included 48 mild episodes, 69 outpatient-treated episodes, 25 requiring emergency department visits, and 50 resulting in hospital admission. Four patients (3.8%) voluntarily withdrew from the study and one patient died (0.9%) (Figure 1).

### 3.2. CAT Score Distribution

**Figure 2:**
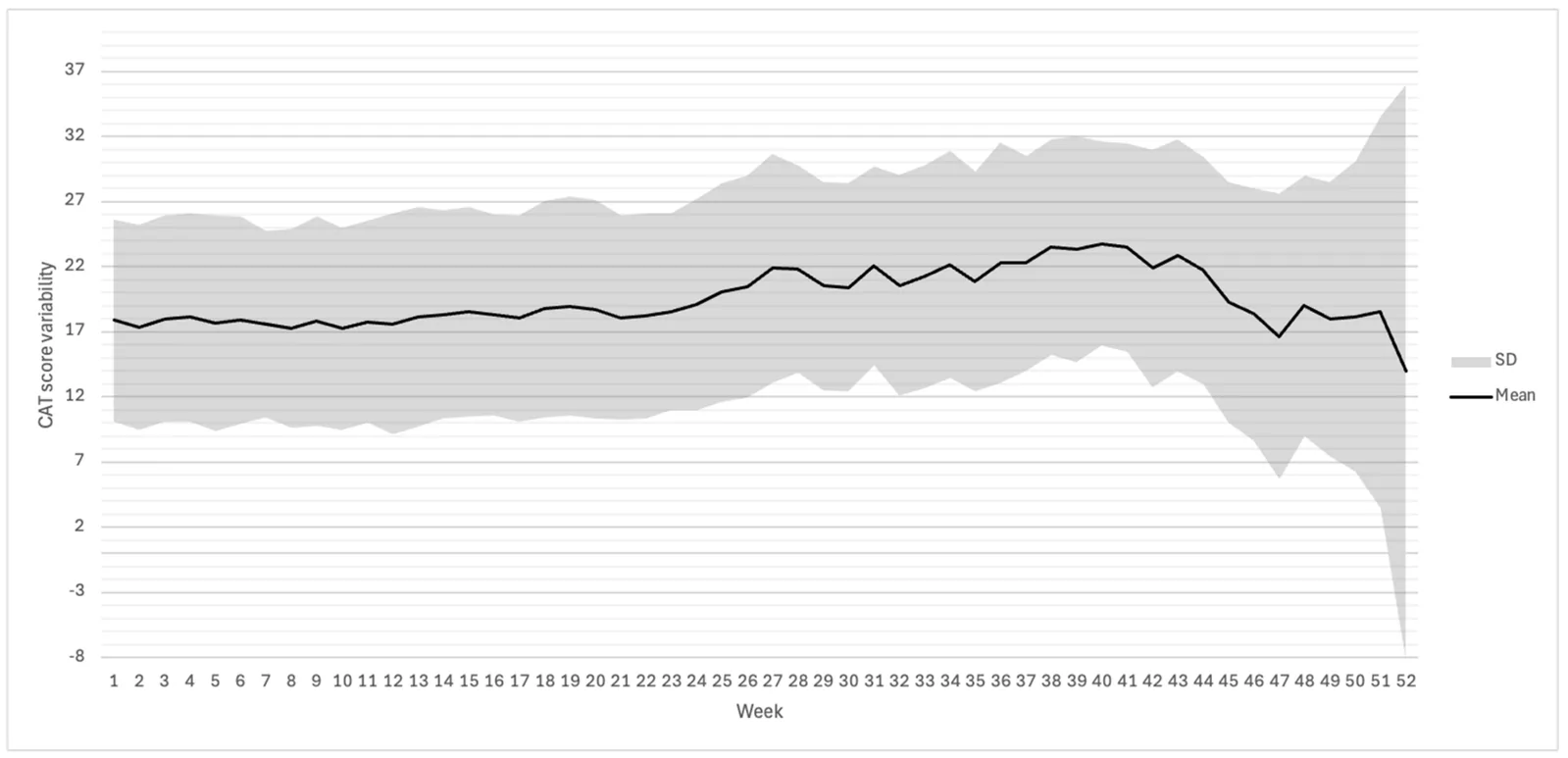
Weekly variations in CAT scores during follow-up.

The median follow-up was 25 weeks (IQR, 20–35). A total of 2885 calls were made, of which 2773 were answered, with 2550 CAT assessments completed, corresponding to 223 incomplete CAT assessments (Figure 1), which represents an average of 23 (IQR 17–31) CAT assessments per patient. The total mean CAT score during follow-up was 18.3 (SD 7.1). Mean CAT scores per patient over time were distributed as follows: 4–10 in 17 patients, 11–20 in 49, 21–30 in 35, and 31–37 in five. The mean variations in CAT score over the follow-up period are presented in Figure 2.

**Figure 3:**
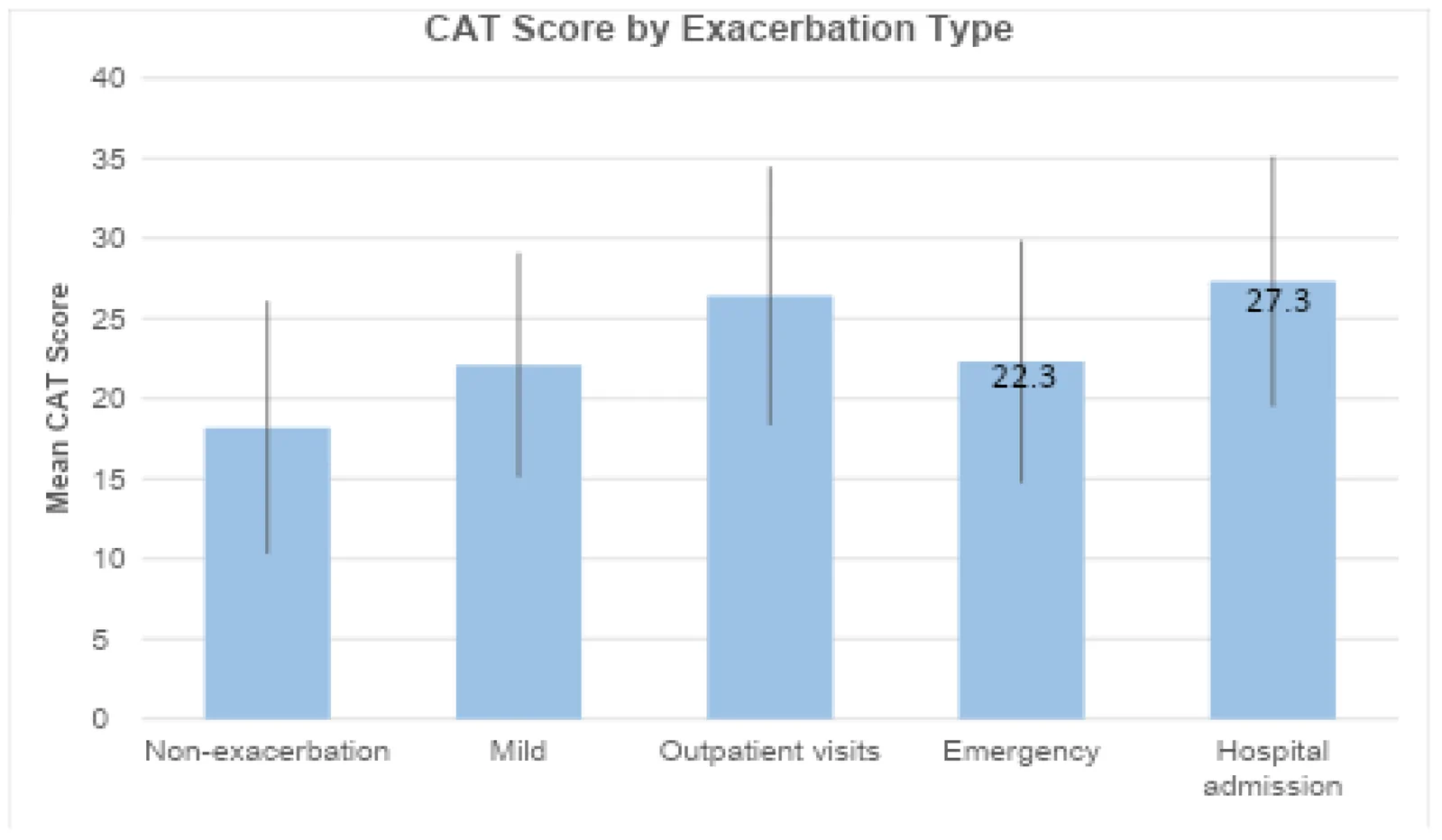
Descriptive analysis based on the mean CAT score throughout follow-up stratified according to the presence and severity of exacerbations (N = 2550 CAT).

Mean CAT scores differed according to exacerbation status and severity during follow-up, ranging from 18.2 (±7.9) in assessments without an exacerbation to 27.3 ± 7.8 in assessments associated with hospital admission. Patients managed through outpatient visits exhibited intermediate CAT scores between these two extremes. These descriptive findings reflect CAT assessments obtained in relation to the exacerbation status recorded during follow-up and should therefore be interpreted as concurrent associations, rather than prospective predictions (Figure 3).

### 3.3. CAT Score Variability

**Figure 4:**
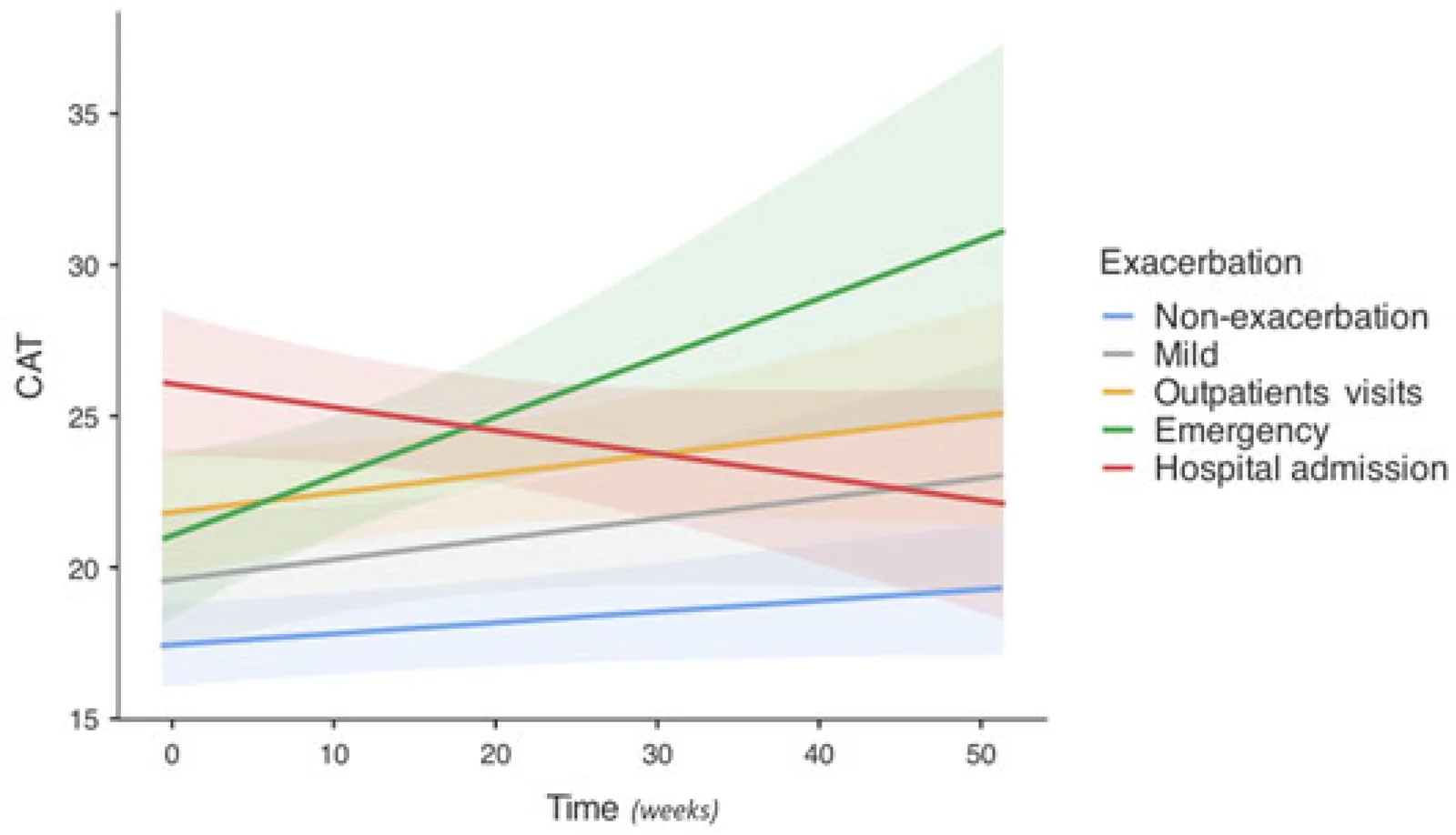
CAT score trajectories over time since study enrollment according to exacerbation type. Gray lines represent individual patient trajectories, while colored lines indicate the estimated average trend for each exacerbation category.

CAT score trajectories differed according to exacerbation status and severity, as indicated by a significant interaction between time and exacerbation type (*p* = 0.021). Compared with non-exacerbation periods, all exacerbation types were associated with higher CAT scores, with progressively larger increases observed for mild exacerbations (β = 2.62; 95% CI: 1.61–3.63; *p* < 0.001), outpatient-treated exacerbations (β = 4.80; 95% CI: 3.97–5.63; *p* < 0.001), emergency department visits (β = 6.07; 95% CI: 4.65–7.49; *p* < 0.001), and hospital admissions (β = 6.87; 95% CI: 5.80–7.94; *p* < 0.001). Over time, CAT scores showed a modest overall increase (β = 0.057 points/week; 95% CI: 0.0003–0.114; *p* = 0.050). However, temporal patterns differed across exacerbation types: hospital admissions were followed by a significant decrease in CAT scores over time (β = −0.11 points/week; 95% CI: −0.20 to −0.02; *p* = 0.015), whereas emergency department visits showed a further increase in CAT scores (β = 0.16 points/week; 95% CI: 0.01–0.31; *p* = 0.036). Thus, the linear mixed-effects model describes longitudinal CAT trajectories according to the exacerbation status recorded at each weekly assessment. Figure 4 illustrates the estimated trajectories of CAT scores over time according to exacerbation type.

### 3.4. CAT Score Variability and Discrimination of Exacerbation Events

**Figure 5:**
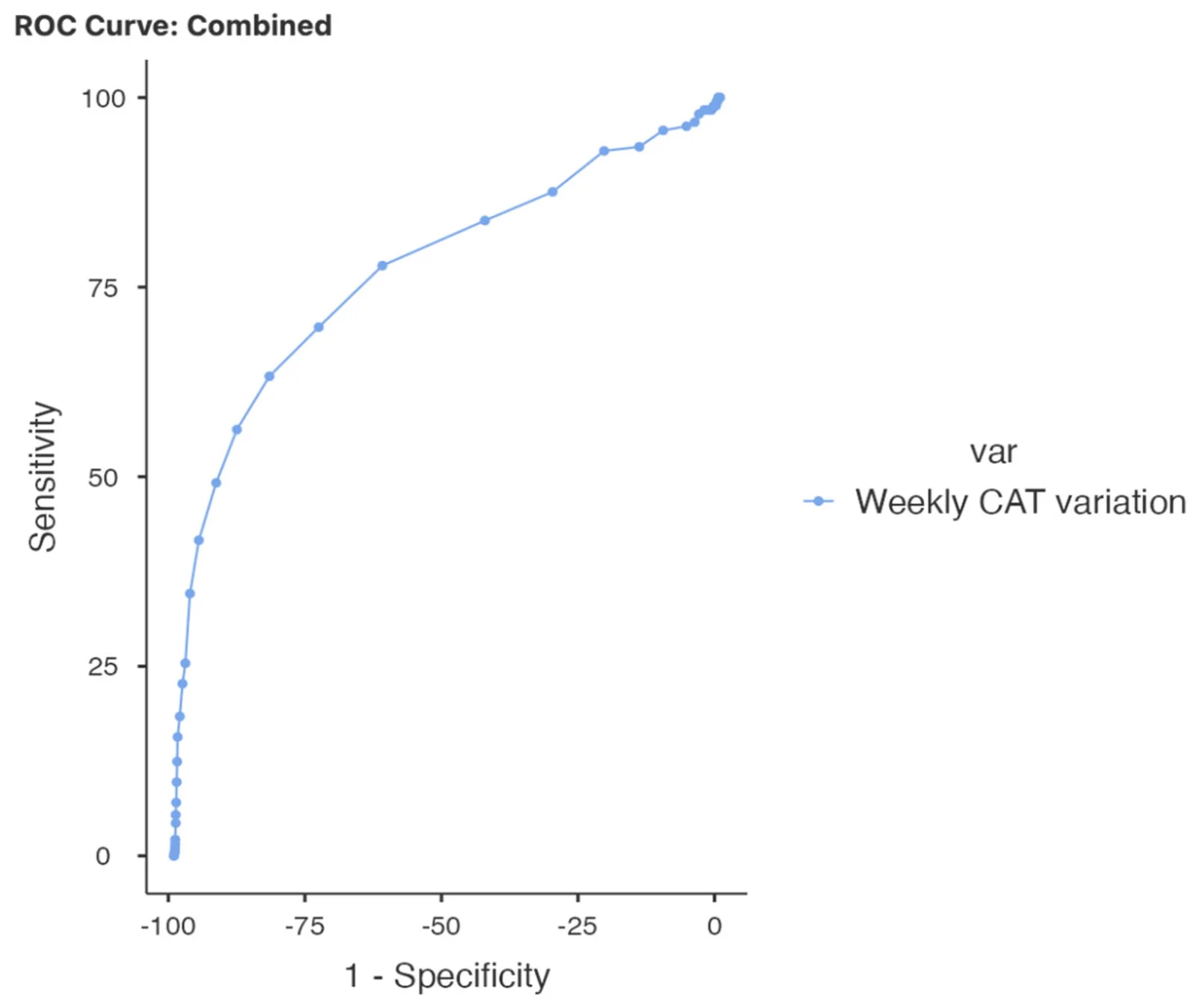
ROC curve for weekly CAT variation in discriminating assessments associated with exacerbation events. The curve illustrates the relationship between sensitivity and 1 − specificity for different cut-off points.

Weekly CAT score variation showed an area under the curve (AUC) of 0.773 for discriminating assessments with exacerbation events. The optimal cut-off point was a 3-point increase, yielding a sensitivity of 63.2%, specificity of 82.5%, Youden index of 0.457, and positive and negative predictive values of 22.8% and 96.5%, respectively (Figure 5). The ROC analysis included 2453 weekly CAT variability assessments from 106 patients, including 185 exacerbation events (7.5% of observations). A patient-level cluster bootstrap sensitivity analysis yielded essentially unchanged results, supporting the robustness of the ROC estimate to within-patient clustering. At the observed exacerbation prevalence, the positive and negative predictive values were 22.8% and 96.5%, respectively.

### 3.5. CAT Score Variability and Exacerbation Events

**Figure 6:**
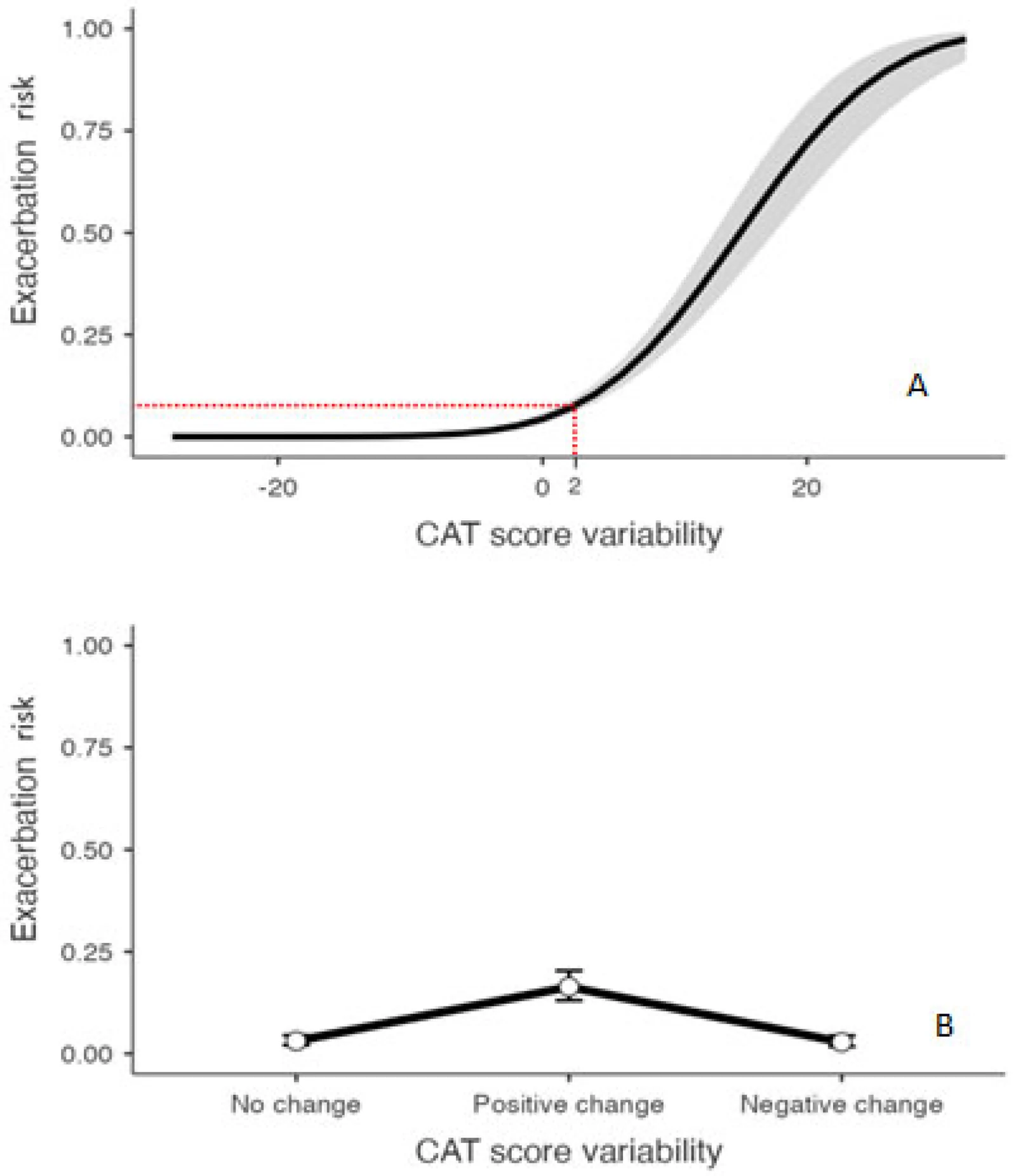
GLM mixed-effects model predicting exacerbation risk based on CAT score variability. (**A**) Absolute CAT score analyzed as a continuous predictor. (**B**) Weekly CAT change categorized as ≥2-point increase, no change (−1 to +1 points), and ≥2-point decrease.

Each one-point increase in absolute CAT score was associated with an 11.1% increase in the odds of exacerbation (OR: 1.11; 95% CI: 1.08–1.13). Additionally, when weekly CAT change was analyzed categorically, an increase of ≥2 points was associated with more than sixfold higher odds of exacerbation compared with no change (OR: 6.30; 95% CI: 4.28–9.27). These findings indicate an association between short-term CAT variability and exacerbation events identified during follow-up (Figure 6).

## 4. Discussion

In this prospective study of patients with COPD and an exacerbator phenotype followed weekly through a virtual assistant, we found a high burden of exacerbations, with more than two thirds of patients experiencing at least one episode during follow-up and one third of them requiring hospital admission. CAT scores were significantly higher during exacerbations compared with stable periods and increased progressively according to exacerbation severity, reaching the highest values during hospitalization. Moreover, a ≥2-point increase in CAT, corresponding to the MCID, was associated with more than sixfold higher odds of exacerbation, while ROC analysis identified a 3-point increase as the optimal discriminative threshold in our study population.

Our cohort represented a population with advanced and clinically unstable COPD, characterized by a median FEV_1_ (%) of 38.8% and a high rate of acute events during follow-up. Accordingly, 69% of patients experienced at least one exacerbation, a proportion higher than that reported in other large prospective cohorts [29,30,31], likely reflecting the greater clinical severity of our population. Although mortality was low, with only one patient dying during follow-up, the relatively short median follow-up of 25 weeks may account for this finding. Nevertheless, the considerable hospitalization burden observed further highlights the severity of the included patients and supports the need for close symptom monitoring.

Our study supports the feasibility of longitudinal symptom monitoring using a voice-based virtual assistant. More than 2500 CAT assessments were successfully collected during follow-up, with more than 96% of answered calls resulting in completed CAT assessments and minimal study withdrawal. These findings suggest that automated telephone-based monitoring may represent a practical and scalable strategy for longitudinal COPD follow-up, particularly in high-risk patients with frequent exacerbations. Importantly, this approach does not require internet access, smartphone use, or advanced digital literacy, which may facilitate implementation in routine clinical practice [26,32,33].

Additionally, the analysis of our data provides insight into the usefulness of the CAT scores obtained by the virtual assistant in stable and exacerbated COPD. There was a significant and clinically relevant difference in CAT scores obtained in calls performed during stable state or exacerbation periods. CAT scores during stable state showed a mean value 18.2, whereas CAT scores during hospitalizations reached a mean of 27.3, which progressively decreased in the weeks after admission. These results are highly consistent with previous studies evaluating CAT during acute exacerbations. García-Sidro et al. [34] found that patients hospitalized for exacerbations of COPD had mean CAT scores close to 25, and that higher CAT values predicted worse short-term outcomes. Similarly, another study on 486 patients with COPD exacerbation reported mean CAT values around 22, with progressive improvement over a period of 4–6 weeks to a mean value of 12 [35].

Worse CAT scores may not only be an indicator of exacerbation status, but in stable patients, may indicate an increased future risk of exacerbations [14,15]. The relationship between CAT variation and exacerbation risk is particularly relevant from a clinical perspective, but fewer studies have evaluated whether short-term changes in CAT are more informative than absolute values. The availability of a virtual assistant offers the possibility of multiple assessments of CAT scores over a relatively short period of time; this paves the way for using the variations in CAT scores for risk evaluation, and/or early identification of exacerbations and the possibility of early management to improve clinical outcomes. The first step must be the evaluation of the variability in CAT scores with weekly determinations. Traditionally, CAT has been interpreted as a cross-sectional measure of symptom burden and health status [11], and there is limited information about the variability in scores with weekly administrations of CAT. The existing studies confirm our results of a marked variability in CAT scores when administered weekly [36,37]. These fluctuations align with the normal variability in respiratory symptoms experienced by most patients with COPD [38]. Miravitlles et al. [39] described significant day-to-day symptom variability even in clinically stable COPD patients, emphasizing that symptom burden should not be considered static; moreover, they also found that factors related to more severe disease and more frequent exacerbations, as in our cohort, were associated with increased symptom variability [39].

Despite this variability, Rassouli et al. [36] showed that weekly CAT measurements can detect clinically relevant changes and may improve early identification of exacerbations, with an 8% increase in the risk of exacerbation per unit increase in CAT score value. In our study, each one-point increase in absolute CAT score was associated with an 11.1% increase in the odds of an exacerbation. Additionally, when assessed categorically, a weekly CAT score increase of two or more points from the preceding assessment (as considered the MCID [28]) was linked to more than a sixfold increase (OR: 6.30) in the likelihood of exacerbation in our cohort.

Although a ≥2-point change has been identified as the MCID and was associated with increased odds of exacerbation in our cohort, ROC analysis identified a 3-point increase as the optimal discriminative threshold. Thus, the ≥2-point change represents a clinically meaningful threshold based on the literature, whereas the 3-point increase represents the optimal discriminative threshold identified in the present cohort. These findings suggest that, although a ≥2-point change is clinically relevant, a 3-point increase provided better discrimination of exacerbation events in our cohort. Of note, the discriminative value of a 3-point increase with weekly administration is very similar to the AUC of 0.789 obtained by Lin et al. [16] with a 2-point change in a six-month period. Furthermore, in line with other studies, this study also confirmed that changes in CAT scores were more strongly associated with exacerbation risk than baseline values alone [16,40].

This study has some limitations. First, it was conducted in a single center with a relatively limited sample size, which may reduce generalizability. Second, the cohort included exclusively exacerbator phenotyped patients with severe airflow limitation, so results may not be directly extrapolated to milder COPD populations; however, the use of such resources for milder or non-exacerbating patients may not be justified. Third, patients willing to participate in automated telephone monitoring may represent a more engaged or adherent population, potentially introducing selection bias. Fourth, the regression analyses were not adjusted for baseline disease severity or treatment, and residual confounding cannot therefore be excluded. Although absolute CAT scores may partly reflect between-patient differences in disease severity, weekly CAT changes capture short-term within-patient symptom variation. Finally, although exacerbations were clinically confirmed, the timing of CAT collection depended on weekly calls, which may have introduced some temporal variability in symptom capture. In addition, 223 CAT assessments were not completed among answered calls, and no imputation was performed. Although analyses used available complete observations, we cannot exclude the possibility of informative missingness, particularly if patients with greater symptom burden were more likely to miss or not complete assessments. Despite these limitations, the prospective design, the large number of repeated CAT measurements, and the use of mixed-effects longitudinal models, which allowed adjustment for repeated observations and inter-individual variability in symptom trajectories, provide strong support for the robustness of our findings.

## 5. Conclusions

Patients with COPD exacerbation are highly compliant with a voice-based virtual assistant. Weekly variability in CAT scores was strongly associated with exacerbation risk and severity in these patients. Short-term increases in CAT scores may help identify patients who warrant closer clinical assessment, but should not be considered a standalone early-warning tool for exacerbation. These findings support the potential benefit of incorporating longitudinal CAT monitoring into clinical practice and digital health programs aimed at improving management of high-risk patients with COPD. Future studies should determine if early action derived from these changes may result in better clinical outcomes.

## Figures and Tables

**Table 1:** Baseline characteristics of the study population (n = 106).

Baseline Characteristics	Total (n = 106)	Exacerbation During Follow-Up (n = 73)	No Exacerbation During Follow-Up (n = 33)	*p*-Value *
Male sex, n (%)	68 (64.2)	44 (60.3)	24 (72.7)	0.216
Age, years, mean (SD)	66.8 (7.9)	65.9 (7.9)	68.7 (7.7)	0.096
BMI, kg/m^2^, mean (SD)	26.3 (5.7)	26.6 (6.2)	25.6 (4.4)	0.398
CAT score, mean (SD)	17.9 (7.6)	18.0 (7.9)	17.3 (7.0)	0.678
mMRC, score, median (IQR)	2 (2–3)	3 (2–3)	2 (2–3)	0.029
CCI, score, median (IQR)	4 (3–7)	4 (3–7)	4 (4–6)	0.592
Emphysema, n (%)	94 (88.7)	66 (90.4)	28 (84.8)	0.509
Chronic bronchitis, n (%)	23 (21.7)	16 (21.9)	7 (21.2)	0.935
Obstructive sleep apnea, n (%)	16 (15.1)	13 (17.8)	3 (9.1)	0.380
Eosinophil count, cells/μL, median (IQR) <100 cells/μL, n (%) ≥100 < 300 cells/μL, n (%) ≥300 cells/μL, n (%)	100 (2.5–300) 29 (27.4) 44 (41.5) 33 (31.1)	100 (0–300) 23 (31.5) 31 (42.5) 19 (26)	200 (100–300) 6 (18.2) 13 (39.4) 14 (42.4)	0.136 0.175
Spirometry post-BD FEV_1_, L, median (IQR) FEV_1_, %, median (IQR) FVC, L, median (IQR) FVC, %, mean (SD) FEV_1_/FVC, %, mean (SD) DCO, %, median (IQR) KCO, %, mean (SD)	1.00 (0.78–1.27) 38.8 (30.1–45.0) 2.24 (1.81–3.09) 69.5 (16.2) 43.9 (11.0) 45.8 (33.9–57.3) 57.0 (21.4)	0.95 (0.78–1.24) 37.8 (29.5–44.0) 2.21 (1.79–2.99) 68.2 (16.3) 43.5 (10.2) 44.5 (33.9–57.1) 55.5 (19.5)	1.04 (0.77–1.34) 39.9 (31.9–47.2) 2.34 (2.01–3.25) 72.2 (15.6) 44.7 (12.5) 47.7 (34.8–59.2) 59.9 (24.7)	0.501 0.235 0.415 0.246 0.597 0.719 0.406
Smoking history Pack-years, median (IQR)	43(35–60)	43 (34–55)	45 (38–60)	0.458
Active, n (%) Former, n (%)	27 (25.5) 79 (74.5)	20 (27.4) 53 (72.6)	7 (21.2) 26 (78.8)	0.499
Treatment SABA, n (%) SAMA, n (%) LABA, n (%) LAMA, n (%) ICS, n (%) OCS, n (%) LTOT, n (%)	6 (5.7) 5 (4.7) 106 (100) 106 (100) 95 (89.6) 2 (1.9) 17 (16)	4 (5.5) 3 (4.1) - - 63 (86.3) 2 (2.7) 12 (16.4)	2 (6.1) 2 (6.1) - - 32 (97.0) - 5 (15.2)	1.000 0.646 - - 0.167 1.000 0.867
Previous-year exacerbations Total Outpatient visits, median (IQR) Emergency department, median (IQR) Hospital admission, median (IQR)	1 (1–3) 1 (0–1) 0 (0–0.75) 1 (0–1)	2 (1–3) 1 (0–1) 0 (0–1) 1 (0–1)	1 (1–2) 1 (0–1) 0 (0–0) 0 (0–1)	0.063 1.000 0.098 0.086

* Footnote: Comparison of exacerbated vs. non-exacerbated groups during follow-up. SD: standard deviation; BMI: body mass index; mMRC: modified Medical Research Council; IQR: interquartile 25–75%; CCI: Charlson comorbidity index; BD: bronchodilator; FEV_1_: forced expiratory volume in one second; FVC: forced vital capacity; DLCO: diffusing capacity of the lungs for carbon monoxide; KCO: carbon monoxide transfer coefficient; SABA: short-acting beta2-agonist; SAMA: short-acting muscarinic antagonist; LABA: long-acting beta2-agonist; LAMA: long-acting muscarinic antagonist; ICS: inhaled corticosteroid; OCS: oral corticosteroid; LTOT: long-term oxygen therapy.

## Data Availability

The data presented in this study are available on request from the corresponding author Data available by request.

## Abbreviations

The following abbreviations are used in this manuscript:

CAT	COPD Assessment Test
COPD	Chronic Obstructive Pulmonary Disease
SD	Standard Deviation
THM	Tucuvi Health Manager
FEV_1_	Forced expiratory volume in 1 s
FVC	Forced vital capacity
DLCO	Carbon monoxide diffusion capacity
MCID	Minimum clinically important difference
SABA	Short-acting beta2-agonist
SAMA	Short-acting muscarinic antagonist
IQR	Interquartile range
ROC	Receiver operating characteristic
AUC	Area under the curve
